# Defective Oligodendroglial Lineage and Demyelination in Amyotrophic Lateral Sclerosis

**DOI:** 10.3390/ijms22073426

**Published:** 2021-03-26

**Authors:** Elisabeth Traiffort, Séverine Morisset-Lopez, Mireille Moussaed, Amina Zahaf

**Affiliations:** 1 Diseases and Hormones of the Nervous System U1195 INSERM, Paris Saclay University, 80 Rue du Général Leclerc, 94276 Le Kremlin-Bicêtre, France; amina.zahaf@inserm.fr; 2 Centre de Biophysique Moléculaire, UPR 4301 CNRS, Orléans University, INSERM, rue Charles Sadron, CEDEX 02, 45071 Orleans, France; severine.morisset-lopez@cnrs-orleans.fr (S.M.-L.); mireille.moussaed@cnrs-orleans.fr (M.M.)

**Keywords:** oligodendrocyte, demyelination, axonal degeneration

## Abstract

Motor neurons and their axons reaching the skeletal muscle have long been considered as the best characterized targets of the degenerative process observed in amyotrophic lateral sclerosis (ALS). However, the involvement of glial cells was also more recently reported. Although oligodendrocytes have been underestimated for a longer time than other cells, they are presently considered as critically involved in axonal injury and also conversely constitute a target for the toxic effects of the degenerative neurons. In the present review, we highlight the recent advances regarding oligodendroglial cell involvement in the pathogenesis of ALS. First, we present the oligodendroglial cells, the process of myelination, and the tight relationship between axons and myelin. The histological abnormalities observed in ALS and animal models of the disease are described, including myelin defects and oligodendroglial accumulation of pathological protein aggregates. Then, we present data that establish the existence of dysfunctional and degenerating oligodendroglial cells, the chain of events resulting in oligodendrocyte degeneration, and the most recent molecular mechanisms supporting oligodendrocyte death and dysfunction. Finally, we review the arguments in support of the primary versus secondary involvement of oligodendrocytes in the disease and discuss the therapeutic perspectives related to oligodendrocyte implication in ALS pathogenesis.

## 1. Introduction

Amyotrophic lateral sclerosis (ALS), also known as Charcot’s disease, is a progressive and late-onset neurodegenerative disease that primarily targets motor neurons leading to fatal paralysis [1]. The first steps include muscular weakness in the arms or legs, which evolves to muscular atrophy, eventually leading to respiratory muscle failure and death. A cognitive decline also progressively occurs. In 1869, the description reported by Charcot in the lateral part of the spinal cord reflected the axonal loss of upper motor neurons descending from the brain and contacting in a direct or indirect manner the lower motor neurons located in the spinal cord.

With an incidence of about 2 per 100,000 people per year, the disease comprises 95% of sporadic cases (sALS) and only 5% of familial forms (fALS) [2,3]. Molecular genetic approaches have been widely applied to ALS research. Genome-wide association studies and “next-generation” sequencing have contributed to delineate that around 40–55% of the genetic causes of fALS can be accounted for by variants in known ALS-linked genes [4]. More than 50 potentially causative or disease-modifying genes have been identified.

However, pathogenic variants in superoxide dismutase 1 (SOD1), hexanucleotide repeat expansions in chromosome 9 open reading frame 72 (C9ORF72), fused in sarcoma/translocated in liposarcoma (FUS), and transactive response DNA binding protein 43 (TDBP43) have been the most widely studied [5]. In sALS cases, diagnostic advancements have only contributed to explain a small part of cases, while the etiology remains unexplained in more than 90% of patients [6]. ALS is characterized by a sexual dimorphism, namely a higher prevalence in males than in females [7] although the ratio of men to women has tended to decrease for the last decades [8,9]. A viral etiology has also been suggested via the detection of human endogenous retrovirus-K detected in neurons of a subpopulation of ALS patients [10,11].

The dramatic and fatal evolution of the disease cannot be halted by any therapeutic approach as shown by the inability of most molecules to alleviate the symptoms, with the exception of Riluzole that slightly increases the lifespan of patients [1,12,13] and whose mechanisms of action have been mostly attributed to the inhibition of glutamatergic transmission and modulation of sodium channel function [14,15]. On the other hand, Edavarone, only approved in 2017, is a free radical scavenger that is believed to act as a neuroprotective agent by reducing oxidative stress and modulate ALS progression during the early stages [16].

Even though alpha motor neurons and their axons in skeletal muscles are the best-characterized target of the degenerative process, other cells are also affected such as Renshaw cells [17], corticospinal neurons [18], parvalbumin-expressing neurons [19], proprioceptive sensory neurons [20], and also glial cells including astrocytes and microglia. The involvement of glial cells in the pathogenesis of ALS was reported about 20 years ago via the study of the transgenic mouse model overexpressing the human mutant of SOD1 [21]. Although mutant SOD1 mice recapitulate the human pathology until the fatal motor neuron degeneration, the latter was found to be non-cell autonomous. Indeed, the expression of the mutant SOD1 in non-neuronal cells contributed to neuronal death [22] and the conditional deletion of mutant SOD1 in astrocytes or microglial cells significantly reduced the progression of the disease [23,24]. In parallel, pathological alterations have been reported in the white matter at a higher level than abnormalities observed in motor neurons [25]. These alterations occur at early stages of ALS before the first clinical symptoms [26] and they are also detected in various rodent models of the disease [27,28,29,30].

In the present review, we propose to highlight the recent advances regarding the involvement of oligodendroglial cells in the pathogenesis of ALS. We will present the oligodendroglial cells, the process of myelination, and the tight relationship between axons and myelin. Oligodendroglial and white matter abnormalities as well as the chain of deleterious events and molecular mechanisms associated with these abnormalities will be described. Finally, we will discuss the therapeutic perspectives provided by the current data involving the oligodendrocyte in the pathogenesis of ALS.

## 2. The Oligodendroglial Cells and Myelination Process

### 2.1. Oligodendrocyte Generation

Responsible for axon myelination in the central nervous system (CNS) and indispensable for its normal function, oligodendrocytes are generated in the developing mouse forebrain from oligodendrocyte precursor cells (OPCs) produced in three waves occurring from the embryonic day 12.5 (E12.5) until a last perinatal wave. The last wave arises from Emx1+ progenitors located in the dorsal subependymal area bordering the lateral ventricles and gives rise to the bulk of myelinating oligodendrocytes leading to active myelination of the forebrain during the first three postnatal weeks [31]. In the developing spinal cord, OPCs arise as two embryonic waves. The first one at E12.5 gives rise to ventral OPCs, which emerge from the same progenitor domain (called pMN) as motor neurons (for review, [32]). The second one at E15.5 is called the dorsal wave. Although only few data are available in the human brain, OPCs were reported to first appear approximately at 9 weeks of gestation, to proliferate until 18 weeks, and to start their differentiation around 27 weeks [33,34,35].

**Figure 1:**
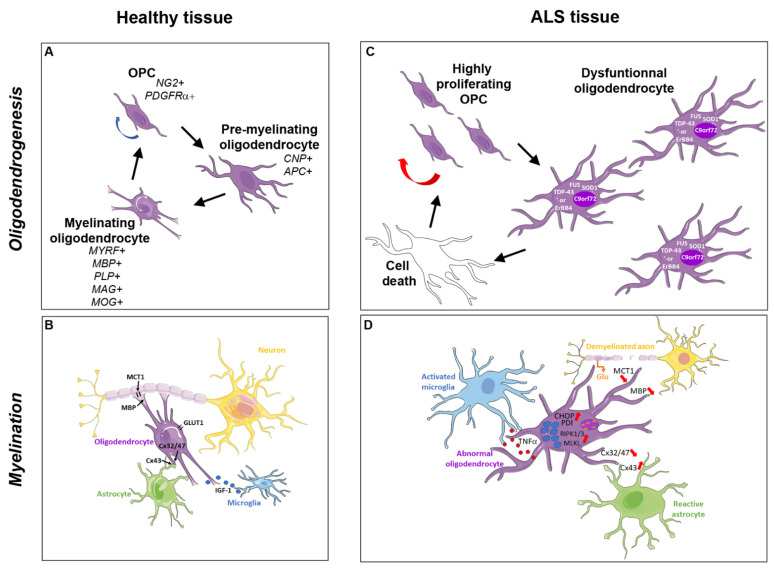
**Pathological oligodendrogenesis and myelination in amyotrophic lateral sclerosis (ALS) disease.** (**A**) In the healthy central nervous system (CNS), oligodendrogenesis comprises the differentiation of oligodendrocyte progenitor cells (OPC) into pre-myelinating oligodendrocytes, which mature into myelinating cells. Specific markers of each phenotypic step are indicated. The blue arrow indicates the proliferation of OPCs. (**B**) Myelination of neuronal axons by oligodendrocytes takes place in the presence of microglial and astroglial cells, which are known to participate namely in the differentiation of OPCs (via the secretion of IGF-1) and the supply of metabolic nutrients (via gap junctions enriched in channel forming proteins connexin, Cx) and lipids for myelin sheath production. (**C**) During ALS disease, oligodendrogenesis is characterized by the increase of OPC proliferation, which gives rise to a higher number of differentiated oligodendrocytes. The latter are dysmorphic and display various protein aggregates in their cytoplasm including (TDP-43 or ErBB4, SOD1, FUS) or accumulate abnormal dipeptide repeat proteins (C9orf72) in their nucleus. These abnormal oligodendrocytes are unable to mature into myelinating cells and ultimately die. In response to cell death, OPC proliferation is highly increased (red arrow). (**D**) Cellular and molecular mechanisms involved in ALS pathology include axon demyelination and degeneration that may occur as the primary or secondary event compared to the death of oligodendrocytes. Both glutamate excitotoxicity (orange broken arrow) from the degenerating neurons and the dysregulation of protein folding leading to abnormal aggregates (blue and orange ellipses) lead to endoplasmic reticulum stress resulting into the increase of the ER stress apoptotic mediator CHOP and into the activation of the unfolded protein response (UPR) associated with the specific expression of select UPR target genes, such as PDI (protein disulfide isomerase). At the level of oligodendrocyte processes, the most abundant myelin protein MBP and the monocarboxylate transporter 1 (MCT1) required for lactate release to the axon are both decreased. The altered communication between the oligodendrocyte and the neighboring astrocytes (green) related to changes in connexin (Cx) expression as well as the pro-inflammatory microglia (blue) namely secreting the cytokine TNFα and triggering the RIPK1/RIPK3/MLKL signaling pathway, both participate in oligodendrocyte dysfunction and degeneration, respectively.

In the murine brain, early oligodendroglial differentiation is mediated by the down-regulation of Sox2 and the up-regulation of Sox 10, Olig1, and Olig 2. At early stages, human and murine cells also express platelet derived growth factor receptor α (PDGFRα) and NG2 (Figure 1A). The transition of OPCs to post-mitotic oligodendrocytes is characterized by chromatin condensation with heterochromatin formation, upregulation of selected microRNAs, and silencing of genes involved in cell proliferation. Loss of PDGFRα/NG2, and increased expression of 2’,3’-Cyclic nucleotide 3’-phosphodiesterase (CNP) and adenomatous polyposis coli (APC), together with Olig 1 and Olig 2, are characteristic of pre-myelinating oligodendrocytes. Finally, myelination triggered by myelin regulatory factor (MYRF) expressed in post-mitotic oligodendrocytes, is accompanied by increased expression of myelin basic protein (MBP), myelin-associated glycoprotein (MAG), proteolipid protein (PLP), and myelin oligodendrocyte glycoprotein (MOG) [36,37]. The oligodendroglial cells can establish synaptic-like structures with neurons and communicate via many receptors and ion channels specifically expressed in OPCs or at different stages of oligodendroglial differentiation and maturation [38].

Although myelinating oligodendrocytes are generated rapidly during the early postnatal period, a small proportion of NG2-expressing OPCs remain thereafter present in both gray and white matter in the adult CNS. OPCs constitute an abundant and widely distributed population of cells that retain the capacity to divide and mature into myelinating cells under physiological and pathological conditions throughout the whole life [39,40,41]. According to their region of origin and their expression profile, OPCs can respond to various insults [42,43]. In agreement with the diversity of oligodendrocytes, transcriptomic profiles using high-throughput methods and single-cell RNA sequencing revealed different subpopulations of oligodendrocytes from several brain regions of juvenile and adult mouse brain [44] and in response to spinal cord injury [45]. Gene expression studies have also identified subpopulations of oligodendroglial cells in human adult white matter and fetal brain [35,36,46]. Altogether, these studies support the existence of highly complex functional and physiological states of oligodendroglial cells.

### 2.2. Axonal Myelination

Myelination of axons essential for rapid impulse propagation in CNS vertebrates occurs during the late embryonic and early postnatal development successively in the spinal cord and in the brain. White matter is predominantly composed of myelinated and unmyelinated neuronal axons organized into specific tracts with surrounding glial cells. Myelin is composed of lipids (70–85%) and proteins (15–30%) among which the most abundant are MBP and PLP while minor proteins include MOG, CNP, and MAG [47]. In white matter tracts that comprise around 50% of the human brain, oligodendrocytes enwrap segments of axons with their plasma membranes [48]. MBP is not translated in the cell body of the oligodendrocyte, where it would cause a fatal compaction of the organelle membranes. Instead, MBP mRNA is silenced and transported to the myelin compartment by the RNA transport granule, a complex of proteins, and RNA molecules. For transport to occur, MBP mRNA must be appropriately spliced and contain the so-called A2 response element (A2RE) in the 3’UTR. The A2RE binds hnRNPA2/B1, which triggers the assembly of the mRNA-transport granule. Translation then occurs in the myelin compartment [49]. The initial contact between oligodendrocytes and axons triggers myelin sheath synthesis from the oligodendrocyte processes in position adjacent to the axon. Each segment, called an internode (for its location between unmyelinated gaps called the nodes of Ranvier), expands longitudinally and simultaneously with axon elongation that progressively occurs during body growth. The last step comprises myelin sheath compaction that starts from the outermost layer [50,51]. The whole process that takes several decades in humans [52] leads to the distribution of myelin segments according to diversified profiles along single axons depending on the neuronal identity [53].

Electrical activity derived from the axon is a major regulator of the myelination process. Indeed, activity-dependent release of ATP has been reported to bind the astrocyte purinergic P2 receptor and to induce the release of LIF cytokine, which ultimately stimulates myelination [54]. Moreover, despite their characterization as the myelinating cells of the CNS, the oligodendrocytes are not the only cells implicated in the myelination process. Indeed, astrocytes provide a substantial fraction of the lipids required for myelin synthesis as suggested by the inability for oligodendrocytes to finalize myelination when a critical co-activator of the transcription factor Sterol Regulatory Element-Binding Protein was inactivated in astrocytes [55]. Physical interactions involving connexins present in both astrocytes and oligodendrocytes were reported to be required for myelination (Figure 1B). In addition, astrocytes contacting the nodes of Ranvier are endowed with the capacity to remodel myelin at the level of its thickness or the internodal length in order to optimize the electrical conduction [56]. Besides astrocytes, microglia or at least a specific subset of these cells uniquely found in the neonatal CNS are endowed with myelinogenic activities via the secretion of various molecules such as IGF-1 [57,58] (Figure 1B). How OPCs succeed in integrating signals from the other glial cells starts to be uncovered. For instance, microglia-derived transglutaminase 2 (TG2), found to bind its OPC receptor GPR56 by using the extracellular matrix protein laminin, is required for regular developmental oligodendrogenesis and subsequent myelination [59]. The critical activity of the extracellular matrix in myelination is also supported by the demonstration that extracellular matrix prepared from decellularized human brain tissue promotes the differentiation of induced pluripotent stem cells (iPSCs) into myelinating oligodendrocytes [60].

### 2.3. Oligodendroglial Metabolic Support to the Axon

In the recent years, it has become evident that myelinating cells maintain the long-term integrity and survival of the axons independently of the presence or absence of myelin [61,62,63]. Indeed, besides electrical insulation of axons, the myelin sheath plays an important role in providing trophic and metabolic supports to the latter [64,65]. As compacted myelin limits supply from the extracellular space, oligodendrocyte support to the axons is essential specifically to allow mitochondrial respiration. This support is based on aerobic glycolysis, where pyruvate and lactate are transferred by diffusion into the myelinated axon [66,67]. Diffusion of metabolites through the myelin sheath occurs through a system of cytoplasmic nanochannels via a mechanism involving CNP that organizes the actin cytoskeleton and prevents complete membrane compaction promoted by MBP, which acts as a zipper for compacting myelin [51]. In agreement with this observation, CNP knockout mice display axonal degeneration in myelinated fibers despite the presence of functional myelin whereas MBP knockout mice exhibit no axonal loss despite absence of myelination [61,63]. Moreover, glycolytic support of fast-spiking axons has been reported to be controlled by activity-dependent glutamate release [68] since released glutamate activates N-methyl-D-aspartate (NMDA) receptors expressed by oligodendrocytes, which in turn up-regulate the expression of oligodendroglial cell surface glucose transporter 1 (GLUT1). Subsequently, lactate is released towards the axon via monocarboxylate transporter 1 (MCT1) and captured from the peri-axonal environment by MCT2 transporter expressed by the axon. Together with reminding the astrocyte-neuron lactate shuttle, metabolic support provided by oligodendrocytes is critical since the depletion of MCT1 in oligodendrocytes was found to lead to motor neuron death [66,67,68].

As each oligodendrocyte has the capacity to produce between 20 and 60 myelinating processes and each of the latter displays an internodal length from 20 to 200 µm [69,70], the oligodendrocyte is one of the cell types with the largest surface membrane and therefore an extensive metabolic demand. Thus, though oligodendrocyte turnover is low as indicated by the replacement of 1 out of 300 cells per year in humans [71], the production of myelin is much more dynamic since one cell is able to produce three times its own weight in myelin per day [72,73]. Both the metabolic supply to axons and the metabolic demand for myelination make oligodendrocyte a major player in CNS homeostasis. In addition, the unique mutually dependent unit formed by the oligodendrocyte and the axon can be considered as a major contributor to the physiological function of axonal connectivity. Consequently, oligodendrocytes and myelin are highly vulnerable to energy metabolism deficiencies even though mature oligodendrocytes are equipped with metabolic mechanisms that can overcome metabolic insults including axon–myelin interactions, which are mutually supportive [66,74,75].

## 3. Pathological Inclusions in Oligodendrocytes from ALS Patients and Animal Models

As of 2003, Clement and collaborators revealed that the expression of mutant SOD1 in motor neurons at levels that classically causes disease is not sufficient to trigger all aspects of ALS pathology. Most importantly, non-neuronal cells that do not express mutant SOD1 delay degeneration and extend survival of motor neurons [22]. The use of mice carrying a mutant SOD1 gene that can be deleted from motor neurons led to the conclusion that these cells are essential for disease onset and its early progression. On the other side, decrease of the mutant levels in microglia significantly slowed disease progression [76] while reducing mutant expression in astrocytes delayed microglia activation and consequently also slowed disease progression [24].

### 3.1. TDP-43 Inclusions

Besides astrocytes and microglia now known to be deeply involved in the progression of ALS [76,77], oligodendrocytes also contribute to motor neuron death in ALS. The aberrant localization of TDP-43 to the cytoplasm rather than the nucleus of neurons has been identified as a morphological hallmark of patients with sALS [78] while mutations in the TARDBP gene identified in fALS patients [79] strengthened the hypothesis that this protein may be involved in the development of the disease. Remarkably, among the first data reporting oligodendrocyte contribution to ALS neuropathology, numerous cytoplasmic inclusions immunoreactive for ubiquitin, p62, and TDP-43 have been visualized in oligodendrocytes in various CNS regions of ALS patients [28,30]. Analysis of a brain bank for neurodegenerative disorders including ALS showed that all samples displaying features of a TDP-43 pathology had morphological features similar to typical sALS. All samples exhibited oligodendroglial cytoplasmic inclusions [80]. Similarly, another study revealed TDP-43+ cytoplasmic inclusions in small cells displaying the characteristics of oligodendroglia that were present in the lower motor nuclei and adjacent white matter, in 97% of sALS cases and all SOD1-negative fALS samples [81]. The severity of gray and white matter oligodendroglial TDP-43+ inclusions was reported to correlate closely with neuronal loss [82]. Moreover, a specific MRI sequence (mcDESPOT) sensitive to water pools within myelin led to showing that the reduction in global cognitive scores and executive function was linked to myelin changes with a frontal lobe predominance in ALS patients. The approach also led to distinguishing the more classical ALS forms from the primary lateral sclerosis, a rare and extreme form of ALS, in which widespread cerebral myelin water fraction is decreased independently of disease duration and clinical upper motor neuron alteration [83].

In the same line, the analysis of myelin in the spinal cord from SOD1^G93A^ transgenic rats revealed ultrastructural disorganization of myelin occurring already in the symptom-free period (60 and 93 days of life) and subsequently worsening in paralyzed animals (120 days of life). These anomalies were associated with decrease of both lipids (phospholipids, cholesterol, cerebrosides) and proteins (PLP, MBP) [29]. They were consistent with SAGE analysis of the whole spinal cord and lower brainstem from pre-symptomatic SOD^G93A^ mice indicating altered expression of genes involved in different biological processes including apoptosis, oxidative stress, ATP biosynthesis, myelination, and axonal transport [84]. Proteomic profiling performed on brain-derived exosomes from SOD1^G93A^ transgenic mice demonstrated a loss of MOG compared with exosomes derived from non-transgenic animals [85]. Demyelination as well as decreased expression of genes related to myelin including MBP, Olig1, and Olig2 were similarly observed in a canine model of ALS expressing mutated SOD1 [86].

Besides the detection of TDP-43 cytoplasmic inclusions within oligodendrocytes in the motor cortex and spinal cord of ALS patients [80,87,88,89,90], similar inclusions of this protein normally involved in various aspects of RNA processing and thus present in the cell nucleus have been observed in different animal models of the disease including the mutant SOD1 mice. Detailed analysis of TDP-43 distribution in the spinal cord at different stages of SOD1^G93A^ animals revealed decreasing amounts of TDP-43 cells from cervical to thoracic spinal cord segments, mostly detected in the ventral compared to the dorsal horn, exclusively in neurons and oligodendrocytes. The amount of TDP-43 positive cells significantly increased at the onset and progression stages of the disease concurrently with the increase of neuron death in the ventral part of the cervical spinal cord [91]. A high level of TDP-43 glial inclusions can also be detected in the C9orf72 mutant, which displays the classical pathology of motor neuron loss, but is nevertheless devoid of the oligodendroglial phenotype [92].

The molecular machinery responsible for cytosolic accumulation of misfolded TDP-43 starts to be uncovered. Indeed, the von Hippel Lindau (VHL)/cullin-2 (CUL2) E3 complex is able to recognize and ubiquitinate misfolded TDP-43 and to promote clearance of the fragmented forms of the protein. However, overexpressed VHL preferentially recognizes misfolded TDP-43 and augments aggregate formation at the juxtanuclear protein quality control center (JUNQ) in agreement with the visualization of cytoplasmic inclusions comprising misfolded TDP-43 and VHL especially in oligodendrocytes in ALS spinal cords. Therefore, the imbalance of VHL/CUL2 may affect oligodendrocytes in ALS by inducing the accumulation of pathogenic proteins, including not only TDP-43, but also mutant SOD1 [93]. Furthermore, the formation of TDP-43 cytoplasmic aggregates examined by time-lapse imaging of rat neural stem cell lines specifically differentiated into neuronal or glial cells including oligodendrocytes revealed growing cytoplasmic aggregates in the transduced cells, that were non-membrane bound, composed of electron-dense fine granular materials of 20–30 nm in diameter, and intermingled with mitochondria and lysosomal vesicles. Aggregate formation was followed by collapse of the cell and the subsequent release of the aggregate as insoluble material in culture media before being incorporated into neighboring neuronal cells, suggesting cell-to-cell spreading [94].

Recently, TDP-43 was found to be required for oligodendroglial physiological functions. Its selective deletion in mature oligodendrocytes in mice revealed the development of progressive neurological symptoms leading to early lethality associated with reduction in myelination. This reduction was proposed to be related to both cell autonomous RIPK1-mediated necroptosis of mature oligodendrocytes and TDP-43–dependent decrease in myelin gene expression. In addition, increased proliferation of OPCs was able to replenish the loss of mature oligodendrocytes, specifically in the white matter. However, degeneration of spinal cord motor neurons was remarkably absent, thus excluding apparent toxicity of oligodendrocyte TDP-43 loss on motor neurons [95].

### 3.2. FUS Inclusions

FUS-containing oligodendroglial cytoplasmic inclusions have been also detected in the CNS of ALS patients exhibiting mutations in the FUS gene. The amount of such inclusions was correlated with disease onset [27]. Analysis of the different forms of the mutant protein in ALS-FUS cases allowed the identification of two distinct clinical and neuropathological patterns with neuronal cytoplasmic inclusions found in early-onset cases in contrast to glial (mainly oligodendroglial) cytoplasmic inclusions detected in late-onset cases [96]. A FUS mouse model expressing cytosol-localized FUS generated by deleting its nuclear-localization signal displayed a notable reduced expression of myelin-related genes in the spinal cord [97] while a mouse model expressing wild-type human FUS mimicking its endogenous expression pattern and level within the CNS developed a progressive albeit mild motor phenotype, but no apparent FUS aggregates. Transcriptomic analysis nevertheless identified expression changes in a small set of genes with preferential expression in neurons and OPCs further suggesting that dysfunctions in the oligodendrocyte lineage cells may also contribute to the observed phenotypes [98]. In the same line, the generation of a novel conditional knockout in which FUS is selectively depleted in oligodendrocytes revealed increased novelty-induced motor activity and enhanced exploratory behavior, both reminiscent of some manifestations of frontotemporal lobe degeneration known to be associated, like fALS, with FUS mutations in humans. Most importantly, the work discovered a novel role of FUS in controlling myelin deposition across all myelinated axons and the selective increase in myelination of small caliber axons suggesting that both myelin ensheathment and axon wrapping are increased in the absence of oligodendroglial FUS. The phenotype was not associated with changes in oligodendrocyte lineage progression, since the densities of OPCs and oligodendrocytes were not modified. From a molecular point of view, the phenotype appeared to be associated with activation of the PI3K/Akt pathway and to a greater expression of the rate-limiting enzyme for cholesterol biosynthesis, the 3-hydroxy-3-méthyl-glutaryl-coenzyme A reductase (HMGCR), via transcriptional activation mediated by Fus direct binding to HMGCR transcript [99].

### 3.3. SOD1 Inclusions

SOD1-containing cytoplasmic inclusions have been also observed in the periaxonal oligodendroglial cytoplasm of SOD1^G93A^ mutant mice analyzed by immunoelectron microscopy [100]. Misfolded SOD1 in the form of granular aggregates was regularly detected in the nuclei of ventral horn glial cells including oligodendrocytes in ALS patients carrying or lacking SOD1 mutations [101] as well as in oligodendrocyte cultures derived from fALS or sALS patients presenting mutations in the SOD1 or TDP-43 genes [102].

### 3.4. ErBB4 Inclusions

Another identified causative gene for autosomal dominant, late-onset fALS, designated as ALS19 and encoding ErBB4, is also at the origin of oligodendroglial inclusions [103]. Like TDP-43, ErBB4 is an important regulator of oligodendrocyte development and maturation controlling myelin formation by oligodendrocytes in the CNS [104,105]. Patients with pathogenic mutations showed typical clinical features of ALS, clinically undistinguishable from those observed in patients with sALS. Functional analysis of mutations at the ALS19 locus revealed reduced autophosphorylation capacity of the ErBB4 protein upon stimulation with NRG-1, suggesting that the disruption of the NRG–ErBB4 pathway may cause motor neuron degeneration. The loss of ErBB4 cytoplasmic immunoreactivity in motor neurons in the anterior horns of the spinal cord was correlated with the severity of motor neuron loss, or with alteration of subcellular localization towards a nucleolar or nuclear localization. An ectopic oligodendroglial ErBB4 immunoreactivity could also be detected. Interestingly, the expression of ErBB4 and TDP-43 appeared to be mutually exclusive in oligodendrocytes [106].

## 4. Degenerating and Dysfunctional Oligodendrocytes in ALS Patients and Animal Models

After the first descriptions of the existence of aberrant protein aggregates in oligodendrocytes, independent groups successively reported complementary data that accurately described oligodendrocyte dysfunction, progressive gray-matter demyelination, and reactive changes in OPCs observed in the motor cortex and spinal cord of both sALS and fALS patients as well as in SOD1^G93A^ mutant mice [67,89,107] (Figure 1C,D).

### 4.1. Reduction of MCT1 Expression

In agreement with the hypothesis that the aggregates may disrupt the active transport and/or the free diffusion of metabolites such as lactate, from the oligodendrocyte to the axon by potentially impairing myelin channel functioning, a first work reported the selective reduction of MCT transporters in the motor cortex of ALS patients [67]. MCT1 decrease was also observed in rodent and canine models of the disease [67,86]. In mouse SOD1 mutants, MCT1 transcripts were downregulated in the ventral horn gray matter of the spinal cord derived from early symptomatic and end-stage animals. The reduced MCT1 reporter expression was not related to a lower number of oligodendroglial cells, since mature oligodendrocytes were unchanged. Moreover, Sh-RNA-mediated downregulation of MCT1 specifically in oligodendrocytes in the optic nerve and corpus callosum induced a dramatic increase in the number of degenerating axons indicating that oligodendrocyte MCT1 was critical for axon survival [67].

### 4.2. Degeneration and Accelerated Turnover of Oligodendroglial Cells

In parallel, further investigations revealed that oligodendrocyte lineage cells are targets of the disease in both human patients with ALS and mutant SOD1 mice. In the transgenic SOD1^G93A^ animals, oligodendrocytes clearly degenerate and die, even before motor neuron loss is evident as shown via immunostaining approaches and genetic fate tracing of oligodendrocytes using the inducible Cre recombination under the PLP promoter. The process that clearly intensified with disease progression was mostly observed in the ventral gray matter and at a lower extent in the central white matter.

Oligodendrocyte degeneration was associated with abnormal morphology including enlarged cytoplasm and elongated reactive processes, cleaved caspase-3 immunoreactivity, chromatin condensation, and the existence of clusters of microglial cells surrounding them. However, the oligodendrocyte number remained unexpectedly constant during disease progression. Therefore, it was proposed the existence of a compensatory production of new cells arising from NG2-expressing OPCs scattered throughout the whole CNS and comprising the main source of new myelinating oligodendrocytes upon a demyelinating event. An increase in proliferating NG2+ cells was observed during the symptomatic phase of the disease in the SOD1^G93A^ mice. The genetic tracing of OPCs using the PDGFRα promoter allowed the detection of a higher proportion of OPCs differentiating into oligodendrocytes [89] while the genetic tracing of differentiated oligodendrocytes using the PLP promoter indicated no difference in the total number of oligodendrocytes in the control or mutant SOD1 mice, but a higher production of new oligodendrocytes in the mutant [108,109]. The increased proliferation and differentiation of OPCs, which themselves exhibited some hypertrophy, reflected their activation selectively in brain regions where motor neurons degenerated [89,107].

### 4.3. Regeneration of Dysfunctional Oligodendrocytes

Besides the abnormal morphology of the newly generated oligodendrocytes, myelin sheaths presented altered lipid and protein composition even before symptom onset. Moreover, the new oligodendrocytes were notably observed close to the degenerating axons suggesting that the abnormal behavior of NG2-expressing cells was a response to degeneration of oligodendrocytes following motor neuron loss [29,89,107]. The newly generated oligodendrocytes were proposed to exist in a differentiated, but non-myelinating state, possibly related to the lack of appropriate targets, perhaps promoting cell death. Such a continuous cycle of proliferation, differentiation, and death of oligodendrocytes in ALS might consequently accelerate the death of neurons already stressed and lacking metabolic support [107].

Various candidates likely to account for the impairment of OPC maturation have been evaluated in ALS models. Notch1 signaling is one of the mechanisms that regulate OPC differentiation during remyelination by promoting OPC expansion but inhibiting differentiation and myelin formation. However, the conditional deletion of Notch1 in OPCs fails to alleviate oligodendrocyte dysfunction or ameliorate disease outcomes in ALS mice [109]. These data are consistent with the recent revisit of Notch signaling in ALS models indicating that the expression patterns of the activated Notch intracellular domain (NICD) and Notch ligands did not much vary in the oligodendroglial lineage in the spinal cord of symptomatic SOD1^G93A^ mice. Thus, the Notch signaling pathway does not appear to contribute much to oligodendroglial dysfunction [110].

A second candidate was the extracellular matrix protein, connective tissue growth factor (CTGF/CCN2), which displays an increased level in the spinal cord from ALS patients and SOD1^G93A^ mice. An in vitro study using Schwann cells as a source of CTFG/CCN2 showed that this molecule inhibits oligodendrocyte myelination and promotes astrocyte reactivity supporting the hypothesis that CTGF/CCN2 may specifically impair oligodendrocyte maturation and inhibit axon myelination in ALS disease [111]. Another study performed in mice expressing a moderate level of the fALS-causing mutation SOD1^G37R^ provided evidence for the possible involvement of other signaling pathways known to regulate oligodendrocyte maturation and myelination. Alterations in RNAs encoding components of these pathways including phosphatidylinositol signaling, FcγR-mediated phagocytosis, and a calcium signaling pathway nevertheless remain to be further investigated [112].

Future works should delineate whether a pro-inflammatory OPC phenotype is present in ALS tissues. This phenotype has been shown to occur in response to inflammatory cues, to promote tissue damage and to block remyelination [113,114]. Thus, this might account for the apparent dysfunction of the newly generated oligodendrocytes. In the same line, the recent finding that alteration of oligodendrocyte heterogeneity in multiple sclerosis, the most common CNS demyelinating disease, may be related to the progression of the disease [113] suggests the requirement for more accurately delineating the phenotype of oligodendrocytes present in ALS tissues.

### 4.4. Abnormal Myelination

The clear reduction of myelination in ALS corticospinal tracts and ventral horn gray matter of the spinal cord was obviously consistent with the finding that the newly generated oligodendrocytes were dysfunctional and failed both in terms of myelin synthesis and trophic support as suggested by the lower expression of MBP and MCT1, respectively [89,107]. Electron microscopy analysis of the ventral gray matter revealed a high percentage of ultrastructurally normal axons exhibiting immature myelin, i.e., a thick layer of oligodendrocyte cytoplasm between the axon and the initial myelin wrap in end stage SOD1^G93A^ mice, suggesting that viable and uninjured axons were remyelinated. Ultrastructural anomalies typical of Wallerian degeneration were also observed together with MBP immunoreactivity more diffuse in the end stage mutant than in the control. Axons with mature myelin displayed a thicker sheath than controls possibly reflecting axonal shrinkage. Myelin debris and immature myelin sheaths could also be detected [107].

### 4.5. Motor Neuron Death Induced by ALS Gene Expression in Oligodendrocytes

Importantly, the genetic deletion of mutant human SOD1^G37R^ from OPCs and their progeny delayed disease onset and prolonged survival, confirming the direct relationship with motor neuron degeneration. SOD1 removal helped to preserve MCT1 expression in some mice at early stages of the disease indicating that expression of mutant SOD1 in NG2+ cells and their oligodendrocyte progeny has a deleterious effect on motor neuron survival. Thus, the progressive loss of gray matter oligodendrocytes as well as the failure to restore these critical cells were proposed to accelerate disease progression in ALS by depriving motor neurons of essential metabolic support [107].

In vitro models of motor neuron and oligodendrocyte cocultures led to consistent results indicating that mouse SOD1^G93A^ oligodendrocytes or human ALS oligodendrocytes derived from multiple genetic and sporadic forms of the disease induce motor neuron death via soluble and insoluble factors that require cell-to-cell contact or very close proximity. Interestingly, the toxic effects can be rescued by reducing SOD1 in OPCs (but not in differentiated oligodendrocytes) except for OPCs derived from cells carrying the C9ORF72 mutations, which seem to be SOD1 independent and thus define a discrete subgroup of ALS patients [102]. In agreement with this observation, oligodendrocytes derived from human pluripotent stem cells obtained from two ALS patients harboring mutations in the C9ORF72 gene did not exhibit impairment of OPC proliferation, maturation, or viability. However, RNA foci could be detected as expected from the expanding RNA repeats present in the mutant. Thus, the presence of the C9ORF72 mutation, the most common manifestation of familial ALS, contributing to 10% of sporadic- and 37% of familial cases, did not confer detrimental effects on maturation or survival of oligodendrocytes. Although the data could not exclude possible impairment of myelination associated with such mutations, they suggested that the different genetic causes of ALS could be heterogeneous with respect to the properties of oligodendrocytes [115]. However, intriguingly, the distribution of C9ORF72 promoter activity was recently found to be enriched in cell types and brain regions that undergo degeneration in ALS suggesting that cell autonomous effects in the altered populations of neurons and oligodendrocytes may nevertheless account for their loss [116].

Still in regard to disease heterogeneity, a yet higher level of complexity was recently provided by transcriptome arrays performed in anterior horn of the spinal cord and frontal cortex from sALS cases that revealed striking regional differences. In the spinal cord, up- and down-regulated clusters were related to inflammation/apoptosis and axoneme structures/protein synthesis, respectively. In the frontal cortex, up-regulated gene clusters involved neurotransmission, synaptic proteins, and vesicle trafficking, whereas down-regulated genes clustered into oligodendrocyte function and myelin-related proteins even though patients were at stages with no apparent impairment [117].

## 5. Molecular Mechanisms Involved in Oligodendrocyte Dysfunction and Degeneration in ALS

### 5.1. Glutamate Excitotoxicity Induced by Motor Neuron Death

Since motor neuron degeneration in both spinal cord and motor cortex is an essential and furthermore necessary component of the disease to confirm diagnosis of ALS, glutamate excitotoxicity was among the first mechanisms implicated in oligodendrocyte pathogenesis in ALS. Indeed, when motor neurons start to degenerate, they release high levels of glutamate [118]. Oligodendrocytes express Ca2+ permeable NMDA receptors, which thus promote an abundant Ca2+ accumulation in the cells leading to their degeneration [119]. However, a series of other mechanisms have been progressively proposed to contribute to oligodendrocyte dysfunction (Figure 1D).

### 5.2. Abnormalities in Protein Homeostasis

Oxidative stress induced by an imbalance of free radicals is also a major contributor to ALS neurodegeneration. It exacerbates homeostatic dysregulation of mitochondria, proteins, genes, and other cellular processes, which are the subject of continuous investigations. The subsequent alteration of protein folding is consistent with the formation of protein aggregates promoted by mutations in ALS-related genes including SOD1, FUS, TDP-43, and ErBB4 as described above. These aggregates cause further endoplasmic reticulum (ER) stress and cell apoptosis as namely suggested for the SOD1 mutant [107]. In agreement with this observation, the analysis of the ER-stress apoptotic mediator CHOP (CCAAT/enhancer binding protein (C/EBP) homologous protein) revealed a marked expression increase in the spinal cords from both sALS patients and ALS transgenic mice (Figure 1D). In the latter, CHOP expression increase could be detected at 14 (symptomatic stage) and 18 to 20 (end stage) weeks. Furthermore, localizations of CHOP were detected in motor neurons and glial cells, such as oligodendrocytes, astrocytes, and microglia, suggesting that the up-regulation of CHOP in various cell types including oligodendrocytes may play pivotal roles in the pathogenesis of ALS [120].

To resolve ER stress and defect in protein homeostasis, cells trigger intrinsic mechanisms called unfolded protein response (UPR) and heat shock response (HSR), two of the major signaling pathways implicated in the restoration of protein homeostasis. In ALS human post-mortem tissues, UPR is activated in both the motor cortex and spinal cord with specific expression of select UPR target genes, such as PDIs (protein disulfide isomerase) observed in motor cortex of sALS cases where these genes strongly correlate with oligodendrocyte markers. In contrast, the ER-associated degradation (ERAD) and HSR genes mostly activated in spinal cord, correlate with the expression of neuronal markers evidencing cell-type-specific contributions. Interestingly, an increase in PDI expression has been reported to occur during active myelination in cortical regions of rats. In line with this observation, a strong PDI expression in oligodendrocytes was observed in SOD1 mice in agreement with abnormalities in plasma and membrane lipid signaling, especially in the early symptomatic stages of ALS [121]. Despite the increased expression of the systems involved in protein homeostasis restauration, a recent metadata analysis of oxidative stress etiology in pre-clinical ALS patients has proposed an insufficient compensatory response to oxidative stress [122].

### 5.3. Oxidative Damage to mRNAs Involved in Myelination

In ALS models, oxidative damage to mRNA has been reported in oligodendrocytes before the symptomatic stage when microgliosis is not yet detected. Among the most vulnerable mRNAs are those involved in mitochondrial electron transport and myelination. Thus, oxidative damage to MBP mRNA likely accounts for reduced MBP expression (Figure 1D) consistent with the myelin defects observed in ALS [123]. The hypothesis that the loss of critical functions in the aggregation-prone genes is involved in myelination defect is in agreement with the rescue of white matter degeneration upon removal of disease causal mutation SOD1 from oligodendrocytes [107] and with the requirement of the normal function of TDP-43 for oligodendrocyte survival and myelination [95]. Of note, many critical mRNAs required for normal oligodendroglial function are locally translated at the myelin sheath. This is true for MBP, MOBP, CAII as well as for several RNA-binding proteins that have been all characterized to be binding partners of proteins involved in ALS pathology and, therefore, might contribute to cellular dysfunction. In agreement with this hypothesis, hnRNP A1 and hnRNP A2/B1, involved in MBP mRNA trafficking, are both binding partners of C9ORF72 (G4C2) repeat RNA foci and TDP-43 [124]. In addition, they display intrinsically disordered domains at their C-terminal end supporting the idea that they are prone to misfolding. Moreover, TDP-43 depletion upregulates specific isoforms of hnRNP A1 specifically susceptible to aggregation [125]. Finally, post-mortem tissues from ALS patients displaying C9ORF72 RNA foci were found to sequester hnRNP H/F required for PLP/DM20 alternative splicing [126] via a mechanism involving their interaction with Quaking in Myelination (QKI) proteins [127].

### 5.4. Deleterious Effects Involving Astrocytes

A growing literature is currently providing evidence for the involvement of extrinsic mechanisms originating in neighboring glial cells and related to the existence of a dual activity for both reactive astrocytes and microglia in inflammatory diseases of the CNS, namely demyelinating diseases (for review [128]). Reactive astrocytes secrete pro-inflammatory molecules promoting the neuroinflammatory process as well as extracellular matrix deleterious for remyelination namely via the formation of aggregates known to prevent OPC differentiation as recently described in several reviews [128,129,130]. Moreover, changes in oligodendrocyte and astrocyte connexin expression have been found to affect oligodendrocyte and neuronal metabolic support in both ALS patients and disease models. Connexins form homotypic or heterotypic gap junctions, namely between astrocytes and oligodendrocytes, through which intercellular communication occurs as attested by exchanges of small molecules namely including lactate and glucose transferred by astrocytes to oligodendrocytes. In mutant SOD1 animals, astrocyte Cx43 is significantly upregulated with disease progression, whereas oligodendrocyte Cx32 and Cx47 are downregulated [131,132] (Figure 1D). The decrease of Cx32 observed earlier as the disease-progressive stage was suggested to be due to enhanced degradation of the protein since Cx32 transcription only started to decrease at the end stage of the disease. Cx47 expression also started to decrease at the disease-progressive stage. However, the decrease was attributed to both decreased transcription and defective transport from the cytosol to the surface membrane and/or increased internalization from the membrane to the cytosol.

All these modifications were particularly evident in the dysmorphic oligodendrocytes accumulating at the anterior horns suggesting a causative relationship between mutant SOD1 and connexin pathology. They were also consistent with the stage-dependent progression of astrogliosis and microglial activation in the anterior horns of the same model. In normal conditions, oligodendrocytes can import glucose through GLUT1 and connexin junctions for glycolysis. The latter can yield sufficient ATP to support oligodendrocyte survival while the aerobic glycolysis product, lactate, can be transferred to axons via MCT1 and used as an energy source for axonal activity. Therefore, loss of membranous Cx47 and Cx32 in oligodendrocytes in mSOD1 ALS model mice may lead to insufficient glucose supply both contributing to oligodendrocyte damage and motor neuron death through energy failure [132]. These finding were supported by the severe demyelination, oligodendrocyte death, and axonal loss in mice lacking Cx32 and Cx47 [133]. The changes in glial network activity through connexin hemichannels likely contribute to the failure of remyelination and eventual death of motor neurons, although further works based on knockout or overexpression models for astrocyte/oligodendrocyte connexins should elucidate the importance of these pathways in the pathogenesis of motor neuron disease.

### 5.5. Deleterious Effects Involving Microglia

Like astrocytes, microglia may be endowed with detrimental effects on oligodendrocytes and myelin via the synthesis of cytokines, chemokines, cell adhesion glycoproteins, or reactive oxygen radicals able to damage axons, myelin, oligodendrocytes, and thus implicated in the initiation and propagation of the inflammatory cascade contributing to demyelination. Several recent publications have addressed the topic [128,134]. However, a specific link between microglia and oligodendrocyte degeneration has been established in ALS via data reporting that the lack of optineurin function in microglia activates an intrinsic cascade that triggers these cells to adopt a RIPK1-dependent pro-inflammatory phenotype (including TNFα secretion) assumed to kill neighboring oligodendrocytes by promoting their necroptosis [93]. Optineurin is a gene for which loss-of-function mutations have been linked to ALS, not for motor neuron death or paralysis, but for other features namely including lower number of axons, abnormally large caliber axons, and signs of thicker, less compact myelin sheaths in the spinal ventrolateral white matter and to a lesser extent in the anterior roots from the age of three weeks [135]. The silencing of optineurin was found to sensitize cultured fibroblasts to necroptosis upon exposure to the caspase inhibitor zVAD-fmk, by involving RIPK1, RIPK3, and the phosphorylated form of the mixed lineage kinase domain-like protein (MLKL), which are all increased in the spinal cord of optineurin knockout mice. In addition, targeting RIPK1/RIPK3 in optineurin and SOD1 mutant mice mitigated the axon-myelin phenotype in both mutants and in addition, delayed onset of motor defects in the SOD1 mutant. Whether the assumed death of oligodendrocytes or a preceding dysfunction of these cells cause the axonopathy still remained unclear [93].

## 6. Oligodendrocyte Degeneration as a Primary or Secondary Event in ALS Pathogenesis

### 6.1. Oligodendrocyte Degeneration as a Primary Event

The observation that oligodendrocyte degeneration occurs earlier than motor neuron loss [89,107] has been the first strong argument for considering oligodendrocyte as the primary event in ALS pathology. In agreement with this hypothesis, alterations in the white matter were found to be more pronounced compared with those in motor neurons and they were detected during the early stages of ALS, prior to the appearance of clinical symptoms [25,26].

A recent work also supported the idea that oligodendrocyte and myelin pathology outstrip neuronal degeneration in some regions by analyzing the extent of inclusion pathology in post-mortem tissues from human sALS and mutant C9ORF72-related ALS (C9ALS) cases [136]. Data revealed a high load of p62 and TDP43 glial inclusions in the prefrontal cortex, precentral gyrus, and spinal cord, which was greater in C9ALS than in sALS cases. Double staining demonstrated that the majority of these inclusions were in oligodendrocytes and the use of immunoblotting led to showing reduced MBP protein levels relative to PLP (a myelin component that relies on protein not mRNA transport) and to neurofilament protein used as an axonal marker in the spinal cord. Such a disproportionate loss of MBP compared to the level of PLP and axonal loss, was in agreement with the likely disruption of MBP mRNA transport. The observation also fully supported that myelin alteration was not secondary to axonal loss however without completely excluding that part of myelin degradation might be secondary to axonal loss [136]. Similarly, although the severity of gray and white matter oligodendroglial TDP-43 inclusions has been reported to mostly correlate with neuronal loss [82], the detection of some gray matter oligodendroglial TDP-43 inclusions in areas without evident neuronal TDP-43 aggregates, neuronal loss, or white matter oligodendroglial TDP-43 pathology suggested that gray matter oligodendroglial involvement could be an early event in the disease process that announced subsequent involvement of neuronal cells. The location of gray matter oligodendrocytes in close proximity to axonal connections of motor neurons was also in support of this hypothesis [82].

Other arguments supporting the primary involvement of oligodendrocytes were provided by the selective deletion of mutant SOD1 protein from the oligodendroglial cells that was found to delay disease onset and improve mouse survival, probably suggesting a key role for oligodendroglial cells in accelerating injury to vulnerable motor neurons. However, the time between symptom onset and death remained unmodified, suggesting that the longer survival time was only related to the delay in disease onset. Furthermore, the role of oligodendrocytes in supporting axonal energy metabolism is also obviously consistent with a major role of oligodendrocytes in the deterioration of motor neurons. In a consistent manner, SOD1 removal from oligodendrocytes led to MCT1 levels comparable to those found in the wild-type mice as well as to a lower level of reactive astrocytes and microglia that may also possibly participate in the delay in disease onset since alterations in astrocytes and microglia are critical for determining disease onset [23,24]. In addition, the generation of transgenic zebrafish selectively expressing G93A mutant SOD1 in mature oligodendrocytes revealed that mutant SOD1 directly induced oligodendrocyte degeneration by disrupting the myelin sheath and downregulating MCT1 subsequently causing spinal motor neuron degeneration. Since in vivo treatment of the mutant zebrafish using potassium channel inhibitors rescued behavioral abnormalities without rescuing MCT1 expression, myelin disruption was nevertheless proposed to induce behavioral abnormalities independently of MCT1 [137].

### 6.2. Oligodendrocyte Degeneration as a Secondary Event

On the contrary, a few other works question the primary involvement of oligodendrocytes in the pathological propagation of the disease.

First, the neuronal expression of mutant SOD1 was found to cause motor neuron degeneration and paralysis in transgenic mice displaying cytosolic dendritic ubiquitinated SOD1 aggregates as a dominant pathological feature. Moreover, the crossing of neuron-specific mutant SOD1 mice with ubiquitously wild-type SOD1-expressing mice led to high wild-type SOD1 aggregation in oligodendrocytes after the onset of neuronal degeneration. The chronologic steps were thus that mutant SOD1 in neurons triggers neuronal degeneration, which subsequently may facilitate aggregate formation in surrounding glial cells [138].

Second, the spread of the pathology into four stages has been initially proposed to occur through corticofugal axonal transmission consistent with the detection of oligodendroglial TDP-43 aggregates along with neuronal inclusions. However, while TDP-43 immunoreactive oligodendrocytes were frequently observed in the white matter under the motor and sensory cortices, no TDP-43 pathology was detected, namely in the white matter along the corticospinal tract of the same tissue samples, [139].

Third, high-throughput RNA sequencing together with translating ribosome affinity purification (TRAP) provided evidence that pathogenesis involves a temporal cascade of cell type-selective damage starting in motor neurons, with subsequent damage within glia that might subsequently drive disease propagation. The data were obtained in mice expressing a moderate level of the fALS-causing mutation SOD1^G37R^. Motor neuron damages included synapse and metabolic abnormalities as well as endoplasmic reticulum stress. Glia-related changes occurred early in astrocytes in genes involved in inflammation/metabolism or standing as targets of the lipid-activated transcription factors PPAR and LXR. Dysregulation of myelination and lipid signaling pathways together with activation of ETS transcription factors appeared in oligodendrocytes only after disease initiation. Gene expression changes were most dramatic in motor neurons (260 significant changes), compared to astrocytes (108) and oligodendrocytes (23 including 14 up-regulated and 9 down-regulated RNAs). Notably, ER chaperones PDI and FKBP9 mRNA levels were much more highly up-regulated in astrocytes and oligodendrocytes compared with motor neurons, suggesting that motor neurons could be intrinsically more vulnerable to unfolded protein accumulation. Unexpectedly, MCT1 decrease previously shown in SOD1^G93A^ mice [67] was not observed in these other SOD1 mutants suggesting that altered levels of MCT1 could not constitute an early damaging factor able to drive initiation of non–cell-autonomous toxicity from oligodendrocytes to motor neurons [112].

Fourth, the transfer of fluorescent wild-type or mutant SOD1 cytosolic proteins occurring first in motor neurons and then in neighboring oligodendrocytes in the gray matter of the ventral spinal cord is still in support of the primary involvement of motor neurons and not oligodendrocytes [140]. The demonstration was based on a retrograde transduction experiment using injection of a recombinant AAV6 expressing wild-type SOD1-CFP into the tibialis anterior muscle in early postnatal mice. Data revealed the appearance of CFP fluorescence first in motor neurons, as early as 11 days post-injection, followed 17–31 days later in oligodendrocytes, consistent with time-dependent transfer from motor neurons to oligodendrocytes. However, since the increase of transferred fluorescent proteins in recipient motor neurons paralleled the development of fluorescence in neighboring mature oligodendrocytes and their morphological changes, it was proposed that oligodendrocytes may serve as a vehicle for subsequent transfer between motor neurons. The precise mechanism of cell–cell transfer was proposed to involve a contact between oligodendrocyte processes and motor neuron cell bodies. However, the exact mechanism of the transfer remains to be investigated [140].

### 6.3. Primary or Secondary Event: A Yet Unanswered Question

It is quite clear that our current knowledge does not allow to conclude on the primary or secondary involvement of oligodendrocytes or motor neurons in ALS. However, a few arguments quite inconsistent with the hypothesis that neurons are primarily involved can be pointed out. Indeed, the increase in oligodendrogenesis reported in the ALS models does not support this hypothesis. First, the production of new oligodendrocytes without target axons to myelinate would be inadequate. Second, as motor axons comprise only a minor proportion of myelinated fibers in the spinal cord and as a single oligodendrocyte can synthesise numerous internodal segments on different axons, healthy non-motor axons should allow oligodendrocyte survival. Third, the observation of ultrastructurally normal axons with immature myelin sheaths in the ventral gray matter of symptomatic SOD1 mutant mice is in favor of the attempt of oligodendrocytes to remyelinate viable axons, rather than to degenerate due to axon loss [107]. For all these reasons, the above data support the idea that oligodendrocyte dysfunction prevents proper axonal support, which may trigger neuron degeneration. Since demyelination is by itself insufficient to induce axon degeneration and has to be associated with oligodendrocyte dysfunction [141,142], the down-regulation of MCT1 is likely one of the associated mechanisms required for neuron degeneration. However, it appears strikingly evident that in the alternative where motor neuron degeneration does not contribute to the initiation of oligodendrocyte pathology, it likely contributes to make surrounding oligodendrocytes more vulnerable namely because of their high energy requirement and damaging oxidative reactions [143]. Further work is needed to better understand the existing discrepancies.

### 6.4. The Hypothesis of the Putative Aberrant Regulation of Olig2 Function

In the spinal cord, an additional hypothesis might be the involvement of an aberrant regulation of Olig2 function, the transcription factor expressed by the progenitor cells that give rise successively to both the motor neurons and the oligodendrocytes in the pMN domain during the embryonic development. In support of this hypothesis, the characterization of ChIP-Seq-based Olig2 target genes in this domain was found to be relevant to the pathogenesis of ALS. Indeed, 740 Olig2 target genes overlapping between the newly generated motor neurons and OPCs have been characterized to be closely related to “alternative splicing” in agreement with increasing evidence indicating that RNA metabolism, including the regulation of transcription and alternative splicing, is profoundly disturbed in ALS. Molecular network analysis also suggested that Olig2 down-regulates a wide range of target genes involved in diverse neuronal and glial functions [144]. Further work should deal with this hypothesis in depth.

Still in the context of embryonic development, the increase in OPC proliferation observed in ALS was found to remind the phenotype observed upon the partial ablation of Sonic Hedgehog (Shh) signaling in the Olig2-expressing progenitors. Shh signaling pathway is well characterized for its contribution to the generation of progenitor cells, which give rise to both motor neurons and OPCs during the embryonic development of the spinal cord. In ShhOlig2−/− transgenic mice, the decrease of OPC production in the ventral spinal cord led dorsal OPCs to populate the entire spinal cord and to take on an altered morphology proposed to be similar to the one found in the vicinity of motor neurons in the SOD1^G93A^ mice. In addition, in the ShhOlig2−/− mice, the morphologically abnormal OPCs failed to participate in the synaptic remodeling of motor neurons in response to motor neuron injury. Therefore, the common characteristic between OPCs in SOD1^G93A^ and ShhOlig2−/− animals is that OPCs are forced to over-proliferate to compensate for either OPC degeneration or reduced ventral OPC production, respectively. Thus, a proposed hypothesis is that OPCs in the ALS models might become progressively unable to contribute to synaptic remodeling of distressed motor neurons [145]. However, the hypothesis again remains to be further investigated.

## 7. Therapeutic Perspectives Provided by Oligodendrocyte Implication in the Pathogenesis of ALS

### 7.1. Available Treatments and Antisense Oligonucleotides

Over the past 20 years, clinical trials in ALS patients have reached limited success likely due to the complex nature of this disease. Various compounds with different mechanisms of action have been studied including Riluzole and Edaravone that constitute the only current treatments for ALS, nevertheless with mild benefits if one considers survival and quality of life (Table 1). Riluzole was approved in 1995 [146] and is endowed with an anti-glutamatergic effect, although the precise mechanism of its action was never fully understood. Edavarone is a novel anti-oxidative agent, believed to be a free-radical scavenger, although again the precise mechanism of its action is not fully elucidated [147]. Masitinib started in the early stages of the disease has been shown to slow down its progression and may likely be approved by the Food and Drug Administration in the near future. This highly specific tyrosine kinase inhibitor blocks the CSF1R and c-Kit pathways, which means the activation of immune cells including mast cells and macrophages, also involved in motor neuron degeneration in SOD1^G93A^. The molecule is currently in Phase III trial [148,149].

Moreover, the most recent therapeutic perspective came from antisense oligonucleotides, which are the first approved drugs to treat spinal muscular atrophy and are currently proposed to be potentially interesting in ALS treatment [150,151]. These short, single-stranded nucleic acids binding to RNA able to alter gene expression are chemically modified to make them able to cross the cell membrane and avoid rapid degradation by nucleases. They are delivered to target cells by using nanoparticles or through conjugation to bioactive ligands or cell penetrating peptides via intrathecal injection, which allows achievement of effective concentrations and capturing by neurons and glia. The first antisense oligonucleotide that reached phase 1 clinical trials targets the SOD1 transcript [152] whereas antisense oligonucleotide targeted to C9ORF72 transcripts have been used in pre-clinical studies [151,153,154,155] (Table 1). In addition, antisense oligonucleotides targeting ataxin-2 have been tested in TDP-43 transgenic mouse models wherein the drug is thought to reduce the propensity of TDP-43 to form pathologic inclusions [156]. Similarly, several pre-clinical investigations on the use of short interfering RNA (siRNA) have been conducted to target ALS genes. However, none have yet reached clinical trials (for review, [157]).

### 7.2. Oligodendrocytes as Targets for Future Treatments in ALS

None of the molecules mentioned above specifically targets the oligodendrocytes. However, several therapeutic avenues are currently investigated in this purpose. They can be divided into a series of approaches requiring further technical or conceptual improvements on one side and the use of promyelinating molecules, which likely stand as the most promising approach in not too a distant future, on the other side. The most important out of these approaches are listed in Table 1.

**Table 1:** **Potential therapies targeting oligodendrocyte defects in ALS.** Abbreviation: ASO, antisense oligonucleotide; CSF, cerebro-spinal fluid; OL, oligodendrocyte; OPC, oligodendrocyte progenitor cell; Tg, transgenic.

Oligodendrocyte Defect.	Potential Therapy	ALS Patients	Animal Models
TDP-43 inclusions	-ASO for Ataxin-2		TDP-43 Tg: extends lifespan and reduces pathology [156]
SOD1 inclusions	-ASO	-Phase 1–2: decreases SOD1 concentration in CSF [158]	
RNA foci C9orf72	-ASO		C9orf72 Tg: protects against ALS [155]
ErBB4 inclusions	-ASO	-	-
Myelin degeneration	Promyelinating molecules -Clemastine (Anti-Histamine H1) -Montelukast (Anti-GPR17) -Bexarotene (RXR-γ agonist) -Tamoxifen	-Mitigates progression [159]	-Prolongs survival in SOD^G93A^ [160] -Rescues SOD^G93A^ OPC differentiation in vitro [161] -Prolongs survival in SOD^G93A^ [162]
Altered trophic support to the axon	Rescue of nutrient flow -Co-factor of carboxylases to produce ATP (Biotin) -Mitochondrial ATP synthesis (RNS60)	-Pilot study: safe and well tolerated [163] -Pilot study: safe and well tolerated [164]	-Prolongs survival in SOD^G93A^ [165]
OL degeneration via: -Glutamate excitotoxicity -Oxidative stress -Neuroinflammation	OL replacement -Anti-glutamatergic (Riluzole) -Anti-oxidative agents (Edavarone) -Modulators of reactive gliosis (Masitinib) (Clemastine) (Cannabinoid receptor 2 antagonist)	-Several Phases 1: delayed disease progression -Approved -Approved -Phase 3 [148,149]	-Prolongs survival in SOD^G93A^ [160] -Limits progression in TDP-43 Tg [166]

#### 7.2.1. Therapeutic Approaches Requiring Improvements

The dysfunction of oligodendrocytes and the cytoplasmic deposits of ALS proteins in these cells (that may be or not one of the causes of the disease) led to propose cell transplantation in order to replenish the lost and/or defective oligodendrocytes. Recently, the transplantation of human glial restricted progenitor cells (hGRPs; Q-Cells^®^) into the lumbar/cervical spinal cord of ALS patients has been proposed. These cells represent one of the earliest precursors within the oligodendroglial and astroglial cell lineage and their usage in ALS therapy might be a beneficial approach resulting in the enrichment of nervous system by healthy donor-derived cells [147]. However, the first trials have not yet provided as promising results as expected, which will require both the accurate optimization of the transplanted cells and the determination of the most appropriate mode of delivery [167]. Moreover, oligodendrocyte replacement should have only restricted effects if it is used alone and will need to concomitantly counteract the deleterious features of the host environment.

The compensation of MCT1 expression decrease and connexin expression dysregulation is currently targeted by molecules able to promote ATP synthesis [163,164]. In the same line, the increase of glucose uptake by the oligodendrocyte might be induced by using pharmacological modulators of NMDA receptors. However, such an approach requires further work in order to improve our current knowledge of the accurate molecular mechanisms involved in the oligodendrocyte metabolic support to the axon [168,169].

Finally, although neither directly linked to oligodendrocyte involvement in ALS pathogenesis nor usable as a possible therapeutic perspective, an amazing observation has reported that myelin antigen-mediated immunization of the SOD1^G93A^ mutant allowed choroid plexus activation and the subsequent recruitment of immunoregulatory cells (including IL-10-producing macrophages and Foxp3 regulatory T cells) in the spinal cord, likely responsible for mitigating disease progression and increasing animal survival [170].

#### 7.2.2. Promyelinating Molecules

Since oligodendrocyte degeneration and death stand as major features of ALS impairing both axonal conductance and metabolic support to neurons, molecules endowed with remyelinating properties might thus restore both defects. The development of such molecules constitutes the current challenge in the therapeutic approach of multiple sclerosis, the most common demyelinating disease of the CNS. In this regard, several targets have been identified during the last years for promoting myelin regeneration in the brain and spinal cord. They include modulators of sphingosine-1-phosphate receptor, muscarinic M1 receptor, histamine H1 receptors, retinoid X receptor γ, or opioid κ-receptors; inhibitors of γ-secretase blocking Notch, leukotriene, or Lingo pathways; modulators of hormone signaling such as androgens, estrogens, tri-iodothyronine, and thyroxine; antipsychotics (quetiapine); antifungals (miconazole); and steroids (clobetasol). Only a few have reached clinical trials of myelin regeneration, which have mostly recruited patients suffering from optic neuritis, a common symptom of multiple sclerosis, often occurring during the early stage of the pathology. The first results indicate some promising outcomes in these patients [171].

Whether outcomes in the optic nerve can be extrapolated to other CNS areas remains unknown. However, targets that are currently considered for multiple sclerosis may be obviously worth exploring in ALS. A few molecules are currently evaluated in the latter (Table 1). For instance, Clemastine, a first-generation histamine H1 antagonist endowed with remyelinating properties in a randomized, controlled, double-blind, cross-over trial in multiple sclerosis patients [172], was tested in SOD1^G93A^ mice and was found to reduce microgliosis, modulate microglia-related inflammatory genes, and enhance motor neuron survival [169] further inciting to consider this molecule in pre-clinical trials as able to target both remyelination and neuroinflammation.

Moreover, the P2Y—like G protein-coupled receptor 17 (GPR17), which has been previously characterized as a good pharmacological target to implement repair and remyelination under several neurodegenerative conditions including multiple sclerosis, was reported to be expressed at a much higher level in the spinal cord from SOD1^G93A^ mice compared to healthy animals. This increase started at the pre-symptomatic stage and was exacerbated at the late symptomatic phase suggesting an altered timing of GPR17 expression. Indeed, GPR17 expression is known to be the result of the complex integration of intrinsic determinants regulating oligodendroglial differentiation with extracellular stimuli acting on the Gpr17 gene. Thus, the maintenance of a high GPR17 expression was proposed to impede proper differentiation of OPCs. Interestingly, the defective differentiation of OPCs isolated from the spinal cord of the SOD1^G93A^ mutant mice was rescued by treatment with the GPR17 antagonist montelukast (a leukotrien receptor antagonist authorized for the management of asthma). This already-marketed, orally available drug may represent a multi-target drug with high translational potential for repurposing strategies namely for ALS management [161]. This work also provided evidence for a discrepancy regarding abnormal differentiation of OPCs isolated from the mouse mutant spinal cord compared to the absence of altered differentiation observed in another work, which analyzed OPCs isolated from the cerebral cortex from the same mutant animals [103]. Again, this observation pointed out the heterogeneity of spinal cord and cortical OPCs, which seems to be strictly related to their CNS region location [173]. Obviously, this discrepancy makes indispensable the consideration of oligodendrocyte heterogeneity in the high-throughput screening of potential promyelinating candidate molecules in the context of ALS.

Although remyelinating molecules seem able to reach pre-clinical and clinical development before the other approaches listed above, they will preferentially have to be endowed with multi-target activities if we consider for instance the involvement of microglia and astrocytes in the inflammatory process contributing to oligodendrocyte degeneration [128,130,174]. A few small molecules endowed with such multi-target properties have been proposed in the context of multiple sclerosis [175,176]. Moreover, even though approaches different from remyelination are still far from a putative clinical application, it seems that their combination with promyelinating molecules would be suitable for both coping with degeneration and neuroinflammation and, promoting regeneration. Most importantly, patient-derived iPSCs will be undoubtedly critical in the identification of the therapeutic approach that should be the most suitable for the ALS phenotype displayed by the patient. Indeed, the generation of oligodendrocytes and other cell types (including motor neurons, astrocytes, and microglia) from iPSCs derived from patients with ALS will be critical as a means to provide a microenvironment that closely resembles the one where the degenerative effects take place. Such a technological approach has been already carried out from fALS caused by mutations in SOD1 or C9ORF72 and was reported as suitable for high-throughput screening of promyelinating small molecules specifically aimed at putative therapeutic perspectives for these familial forms of the disease [177].

## 8. Conclusions

The critical role of oligodendrocytes in ALS is presently indisputable. The degeneration of oligodendrocytes during disease progression together with the increased proliferation of OPCs without full differentiation into mature oligodendrocytes obviously participate in the whole spectrum of damages contributing to neurodegeneration. The failure of metabolic support provided to axons and the high level of demyelination directly related to the defective oligodendroglial lineage should both precipitate the degeneration of axons and death of neurons. Conversely, motor neuron degeneration may also contribute to make surrounding oligodendrocytes more vulnerable due to the high energy requirement and damaging oxidative reactions. The key players of the signaling pathways up to now identified as being implicated in oligodendroglial cell alterations during ALS disease already provide a valuable source of molecular targets to develop new cell type selective therapeutic approaches. In addition, since our current knowledge regarding both glial heterogeneity and cellular communication is conspicuously facilitated by highly performant ’OMIC’ approaches as shown in several other neurodegenerative diseases, one may consider that specific transcriptomic changes in cells displaying toxic ALS-related protein aggregates and in closely neighboring cells should undoubtedly improve our understanding of the oligodendrocyte communication with other glial or neuronal cells as well as their impact on disease progression. Similarly, the emergence of human iPSC models for ALS together with gene editing technology will allow investigation of the intricate crosstalk between the various cell types including oligodendrocytes in the human disease.
